# Medical students’ perceptions and attitudes about family practice: a qualitative research synthesis

**DOI:** 10.1186/1472-6920-12-81

**Published:** 2012-08-21

**Authors:** Anna Selva Olid, Amando Martín Zurro, Josep Jiménez Villa, Antonio Monreal Hijar, Xavier Mundet Tuduri, Ángel Otero Puime, Gemma Mas Dalmau, Pablo Alonso‐ Coello

**Affiliations:** 1 The Sant Pau Biomedical Research Institute (IIB Sant Pau), Barcelona, Spain; 2 Servicio Catalán de la Salud, División de Planificación y Evaluación Operativa, Barcelona, Spain; 3 Institut Universitari d'Investigació en Atenció Primària Jordi Gol (IDIAP Jordi Gol), Gran Via 587 àtic, 08007, Barcelona, Spain; 4 Cátedra UAB-Novartis de Docencia e Investigación en Medicina de Familia, Universitat Autònoma de Barcelona, Barcelona, Spain; 5 Cátedra UNIZAR-Novartis de Docencia e Investigación en Medicina de Familia y Atención Primaria, Universidad de Zaragoza, Zaragoza, Spain; 6 Cátedra UAM-Novartis de Docencia e Investigación en Medicina de Familia y Atención Primaria, Universidad Autónoma de Madrid, Madrid, Spain; 7 Iberoamerican Cochrane Centre, CIBERESP-IIB Sant Pau, Barcelona, Spain

**Keywords:** Family practice, Attitudes, Perceptions, Students, Medical, Qualitative research, Review

## Abstract

**Background:**

During the last decade medical students from most Western countries have shown little interest in family practice. Understanding the factors that influence medical students to choose family medicine is crucial.

**Objective:**

To systematically review and synthesize published evidence about medical students’ attitudes and perceptions towards family practice.

**Methods:**

A qualitative systematic review. The literature search was undertaken in July 2010 in PubMed, EMBASE, Cumulative Index to Nursing and Allied Health Literature (CINAHL), Social Science Citation Index (SSCI), and ProQuest Dissertations & Theses. Two authors independently selected the studies for their inclusion and assessed their quality. The selected studies were thoroughly read. Key themes and categories were identified. A matrix was created for allowing the comparison of each theme across studies.

**Results:**

Ten studies were finally included. Seven broad themes were identified across them: 1) Scope and context of practice was a broad theme comprising linked sub-themes: perception of a varied specialty, broad practice, holistic perspective and flexibility that allows having a family; 2) Lower interest or intellectually less challenging: treating common disease, repetitive, quasi administrative job; 3) Influence of role models, either positive and negative, and society: negative comments from other professionals, peers and family; 4) Lower prestige; 5) Poor remuneration; 6) Medical school influences, being important both the length and quality of the exposure; 7) Post graduate training, where the shorter duration and the lower intensity were perceived as positive aspects. After identifying these seven key themes, were also looked into patterns in the distribution of these themes among studies.

**Conclusions:**

Our qualitative review provides a comprehensive picture of medical students’ attitudes towards family practice in the available literature. In general, although some students find family medicine appealing, it is regarded as a career of low interest and prestige. More research is needed on the influence of role models, medical school and post graduate training.

## Background

Despite a likely high demand for family practitioners in the near future [1], during the last decade medical students from most Western countries have shown little interest in family practice as a career choice [2-5]. The proportion of general practitioners is smaller than the overall number of specialists in most OECD countries (Organisation for Economic Cooperation and Development, 2007) [6]. Being a complex phenomenon caused by multiple factors, the shortage of family physicians has become a concern for many nations.

Understanding the factors that influence medical students to choose family medicine is crucial in order to prevent possible family physicians’ shortage in the future. A previous systematic review [7] showed that older age, lower socioeconomic status or lower parental income or education, rural background, values and knowledge (the belief that primary care is important, low income expectations, absence of plans for a career in research) as well as career intentions at entry to medical school were all factors associated with a higher likelihood of choosing family practice. In addition public ownership of the school has been related positively with the choice of family practice [7]. The most important factors in the decision making process are medical school related factors, specially the stated third and fourth year curricula, the amount of time devoted to family practice, the “hidden curriculum” (created by the opinions and comments of students, residents and faculty), the negative and positive experiences of particular disciplines, the effect of role models, and the physical and professional environments in which education is delivered [7,8].

During the years of medical education, students develop perceptions about the content and characteristics of each specialty. These beliefs have an important role in the students final choice of their speciality [7]. Some studies state that concerns about prestige, low income, and the breadth of knowledge required are associated with a rejection of family practice [7,9].

Several surveys examining the perceptions and attitudes of medical students about family medicine have been conducted previously [9-12]. A number of studies suggest that the medical students’ attitude towards family medicine improves, as they progress in medical school and this may be partially explained by the greater contact with general physicians [12].

Despite the usefulness of surveys and questionnaires in measuring perceptions and attitudes, qualitative research is more suitable when trying to understand the meaning people give to the subject of interest in their situated contexts. To our knowledge, there is no previous systematic review on this topic, adopting a qualitative research perspective. The aim of the present study is to develop a synthesis of the available qualitative studies exploring medical students’ perceptions and attitudes towards family practice.

## Methods

A systematic review and synthesis of qualitative studies using a thematic synthesis approach was conducted [13].

### Selection criteria

To begin, the target population were medical students. The studies of interest focused on the evaluation of their perceptions and attitudes about family practice. Second, we included qualitative studies based on data collected through focus groups; open, structured or semi-structured interviews; or any study which used qualitative methodology (that is, text-based and interpretive). We excluded questionnaires and surveys unless these were part of studies using mixed methods with qualitative research data. Third, we only considered studies written in English, Spanish, Italian, Portuguese or French.

### Identification and selection of relevant studies

The following databases were searched: PubMed, EMBASE, Cumulative Index to Nursing and Allied Health Literature (CINAHL) and Social Science Citation Index (SSCI) from their inception dates until 5th July 2010. We also looked for doctoral theses in ProQuest Dissertations & Theses. Search strategies were developed for each database in collaboration with a librarian and included the following categories: medical students, attitudes, perceptions, general practice, primary health care and family practice (Additional file 1).

Two authors independently assessed all retrieved titles and abstracts, and identified studies that fulfilled the selection criteria. Following this, full-text versions of the chosen papers were obtained and independently examined. Disagreements about inclusion were resolved through consensus and in case of discrepancy by a third author. The quality of the papers was investigated using an adaptation of the Critical Appraisal Skills Programme (CASP) tool for qualitative studies [14].

### Analytical approach

The selected studies were thoroughly read. The type of study, its methodology, how information was collected and what type of analysis was performed for each study was identified. The authors of the studies were contacted to confirm witch methodology and type of analysis was used (Table 1). Key themes and sub-themes were identified. The process of theme searching was dynamic and it did not finish until all the studies were accounted for. Emerging theme definitions and limits were discussed for their development and refinement. A descriptive chart was created for each study including key information such as: author, date, country, methodology, results, quality and limitations (Additional file 2). The initial list of themes was used to create a matrix, derived from an approach described by Miles and Huberman [15], allowing the comparison of each theme across the studies. This matrix was reviewed and refined and the themes were grouped until it was possible to synthesize all the studies. 

**Table 1:** Characteristics of included studies

**Study**	**Country**	**Methodology**	**Type of study**	**Technique**	**Phenomenon of interest**	**Participants**	**Analysis**
**Tolhurst **[16]** 2005**	Australia	Qualitative: phenomenology*	Descriptive*	Focus groups	Factors influencing career interests of medical students.	81 first and final year medical students. (36 male, 46 female). Three universities	Interpretative. Thematic analysis.
**Saigal **[17]** 2007**	Japan	Qualitative: phenomenology*	Descriptive*	Semi-structured interviews (students), informal interviews (academic faculty). Field notes.	Factors influencing medical specialty preference in Japan. Understanding of family medicine, primary care and subspecialty practice.	25 medical students or 3^rd^ to 6^th^ year. (17 male, 8 female). One university	Interpretative. Thematic analysis.*
**Scott **[18]** 2007**	Canada	Qualitative: phenomenology.	Descriptive	Focus groups Individual interviews	Factors influencing medical students regarding a career in family medicine.	33 medical students: end of preclinical years and end of the clinical years. (6 male, 27 female). Three universities	Interpretative. Thematic analysis.
**Thistlethwaite **[19]** 2008**	Australia	Qualitative: phenomenology	Exploratory-interpretative	Semi-structured phone interviews	Factors that influence students and junior doctors to choose or reject a career in general practice.	13 medical students (3 male, 10 female), 5 junior doctors, 5 general practice registrars, 15 general physicians. One university.	Interpretative. Thematic analysis.
**López-Roig **[20]** 2010**	Spain	Qualitative: case based research. Phenomenology	Interpretative	Focus groups Documental analysis	Explore the reputation of and professional identification processes with family medicine practice among students.	48 students: 27 2nd year medical students, 21 6th year medical students. One university	Interpretative. Discursive thematic analysis.*
**Hogg **[21]** 2008**	United Kingdom	Mixed: qualitative interactionist and quantitative.	Exploratory, descriptive and interpretative	Focus groups Questionnaires	Factors influencing medical students regarding a career in general practice.	30 final year medical students after a general practice module: 15 took part in the focus groups.	Interpretative. Framework analysis.
**Edgcumbe **[22]** 2008**	United Kingdom	Qualitative: framework system	Exploratory	Semi-structured interviews. Nominal groups	Views about general practice as a potential career and factors shaping them.	27 final year medical students (7 male, 8 female). 15 interviewed and 12 formed the nominal group.	Interpretative. Framework system.
**Chirk-Jenn 2005 **[23]	Malaysia	Qualitative: interpretative description.	Exploratory	Focus groups	Perceptions of medical students towards primary care and factors that influence them.	33 final year medical students (21 male, 12 female). Two universities.	Thematic analysis.
**Firth 2007 **[24]	United Kingdom	Qualitative: phenomenology	Exploratory	Semi-structured interviews	Views of undergraduate students on their experiences of learning in primary care in a curriculum with a strong community base.	11 medical students from 3^rd^ to 5^th^ course (6 male, 5 female)	Interpretative. Thematic analysis. Grounded Theory.
**Mutha 1997 **[25]	USA	Qualitative: phenomenology	Descriptive	Focus groups, two individual interviews, surveys.	To identify beliefs and values that influence career decisions of medical students.	52 medical students from 4^th^ to 5^th^ course (25 male, 27 female). Three medical schools.	Content and thematic analysis.

*Unclear, not explicitly reported.

## Results

**Figure 1:**
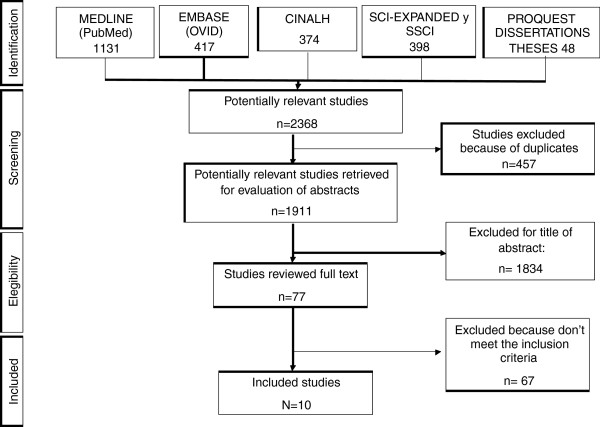
Study inclusion/exclusion process.

From 2368 retrieved studies, 457 duplicates were excluded and 1826 through revision of the title and abstract. Moreover, the full text of 77 papers was evaluated. Finally ten papers were chosen, which fulfilled the inclusion criteria (Figure 1).

The included studies were published between 1997 and 2010 and were all in English. Three studies were from the UK, two from Australia, and one each from Canada, USA, Japan, Malaysia and Spain. Four studies explored views and perceptions of medical students about family medicine [17,20,22,23]. Another four investigated the factors influencing a career choice on family medicine [18-22]. Three others examined the factors influencing medical students’ career interests [16,17,25]. Lastly, one dealt with the views on the experience of learning in primary care in a curriculum with a strong community base [24].

The participants of all the studies were medical students and only six studies included final year university students [17,21-25] and three studies included both, first and final years students [16,18,20]. One study also included junior doctors, general practice registrars and general physicians [19]. The number of participants ranged from 11 to 81 medical students. Five studies used focus groups [16,20-25], three used semi-structured interviews [17,19,24], one used both, focus groups and individual interviews [18] while another one used nominal groups and semi-structured interviews [22]. Two other studies also used questionnaires or surveys [21,25] (Table 1). The overall quality of the studies was high, with the exception of one study being of moderate quality. A descriptive table of each study is available in Additional file 2.

### Themes

Seven broad themes were identified (Table 2).

**Table 2:** List of studies, extracted themes and findings

**Study**	**Scope and context of practice**	**Lower interest or intellectually less challenging**	**Influence of role models and society**	**Prestige**	**Poor remuneration**	**Medical school influences on specialty choice**	**Post graduate training**
Tolhurst *et al.* 2005 [16]	▪Diversity, continuity of care +	▪A lot of paperwork-	▪Negative attitudes from specialist and teachers to general practice-		▪Poor remuneration-	▪Undergraduate experiences influenced depending on GPs’ attitudes.+/ -	▪Less intensity and length of training, less long working hours.
	▪Community and family context +	▪Serious problems are referred to specialists-	▪Family and friends pressure to choose a specialty -				
	▪Use of pre-existing skills +						
	▪Less medical indemnity issues+						
	▪Discomfort assessing the urgency of undifferentiated problems -						
	▪Prefer focus on a particular area of expertise -						
	▪Flexibility and part time work allow having a family +						
	▪Rural practice: practice a lot of skills +, is workload and a lot of responsibility -						
Saigal *et al.* 2007 [17]	▪Holistic perspective.	▪Common disease, easy to treat.	▪Personality of physicians influences on choice.	▪A second career that follows working first in a sub specialty.		▪The length and quality of the exposure	
	▪Treat the entire family.		▪The presence of a physician role model or mentor.			▪The atmosphere	
	▪Community based.						
	▪Long term care.						
	▪Good relation doctor-patient+						
	▪Focused on prevention, triage and medical interviews.						
	▪Home visits.						
	▪Primary consultation before seeing specialists.						
	▪Broad knowledge than specialities.						
Scott *et al.* 2007 [18]	▪Broad scope of practice especially in rural settings+	▪Choosing family medicine seems to limit oneself, especially for high-achieving students-	▪Role models affect the choice +/−	▪Lower prestige.	▪Worries about income during their practice life	▪Little representation of family medicine in the curriculum -	▪The easy of matching with family medicine (−)
	▪Enduring relationships with patients.		▪Negative view by other specialists-	▪Second-choice residency.			▪Shorter and physically less demanding residency (+)
	▪Good lifestyle, flexibility+						▪The culture of the family medicine residency is appealing. (+)
Thistlethwaite *et al.* 2008 [19]	▪Continuity of care+	▪Lack of support.	▪Negative role models.-	▪Family medicine has prestige but decreasing.		▪Medical education mainly hospital based.	
	▪Patient-doctor interaction+	▪Lack of time	▪Negative views of GP expressed by hospital doctors without reasons-.	▪Social status.		▪Having general practice exposure earlier +	
	▪Holistic care+	▪Not intellectually challenging.	▪Negative media coverage-	▪General practice is seen as inferior choice.		▪General practice exposure was more stimulating than expected: needs hand-on experience not just observation.	
	▪Skill mix					▪Sell GP as a great job	
	▪Stimulating and variety+						
	▪Working with people+						
	▪Autonomy+						
	▪Flexible working hours and lifestyle+						
	▪Rural practice: hard work.						
López- Roig *et al.* 2010 [20]	▪Holistic care +	▪Broad and superficial knowledge -	▪Social and academic persuasion for not choosing family medicine.	▪Lost of social role.	▪Lower salaries. ▪Less probability of additional income when practicing in the private sector	▪Undergraduate experiences are significant.	▪The four year residency programme is unnecessary (−).
	▪Special relationship with patients+			▪At the bottom of the medical hierarchy.		▪Almost no exposure to family medicine practice: poor idea of what family medicine practice is.	
	▪The kindest and more tolerant doctors.	▪Repetitive -		▪Unknown status of family medicine as a medical specialty.		▪Exposure to (a few) good family medicine experiences in later training years.	
	▪The largest breath but depthless medical wisdom.	▪Lack of intellectual challenge.		▪Lack of professional recognition.			
		▪Absence of medical “technology”-		▪Lower status and facilities.			
		▪Devalued type of knowledge needed to practice.		▪Population and health care decision-makers do not appreciate Family medicine.			
		▪Quasi administrative -		▪Family medicine is a necessary specialty but undesirable as a career option.			
		▪Elderly patients-					
		▪Gatekeepers of the health care system.					
		▪First medical contact and referrer to specialties.					
Hogg *et al.* 2008 [21]	▪Varied, challenging+	▪Lower level of control over the medical care and have to refer to specialist.-	▪Bad mouthing from family and hospital doctors-	▪Lower status than hospital based careers -		▪Perception of the early experiences as not “real” medicine.	
	▪Preference for a career in hospital settings-		▪Bad mouthing from family			▪Importance of general practice exposure+	
	▪Work outside the medical hierarchy.		▪No attractive media role models -				
	▪The best of both worlds: a GPs with a special interest						
	▪Flexibility +						
	▪Control over financial affairs, working hours and lifestyle +						
	▪A backup career when you want to make your life external to the medicine a priority.						
Edgcumbe et al. 2008 [22]	▪holistic care +/−	▪General practice as a go-between -	▪hospital doctors made derogatory comments about general practitioners and vice versa but it not influenced students’ career choice.	▪Lower status than hospital based specialists -	▪business aspects of running a practice -.	▪The career intentions were influenced by experiences of clinical training.	▪Short, well structured and flexible compared to hospital-based medicine.
	▪variety of conditions + vs monotony –	▪Prefer acute conditions and deal with problems without referral.-		▪The status doesn’t always influences career intentions +	▪the 2003 GP contract impinges on the professional autonomy -	▪This experiences were + or – for some students. (some had negative preconceptions before exposure that decreased with it +)	▪Competition in hospital training is unattractive
	▪anxiety for wanting quick answers in diagnosis –	▪mundane/ repetitive -			▪Well paid or overpaid (particularly at earlier stages of career) +		▪Lack of research +/ -
	▪relationship with patients +	▪administrative work-		▪A second line option after a hospital career-			
	▪feeling part of the community +	▪lack of time -					
	▪public health +	▪low-technology environment-					
	▪concerns in managing risk -☺	▪Professional isolation -					
	▪friendly work environment +						
	▪ work anywhere vs remain in one place after buying into a practice +/−						
	▪flexibility, lifestyle, easy to have a family +						
	▪independence +						
Chirk-Jenn *et al.* 2005 [23]	▪holistic, comprehensive +	▪bored by repetition of common illnesses –	▪opinions from colleagues and seniors influenced their perceptions			▪disparity between training and practice: what was taught in their classes was not practised: time pressure. lack of support and difficulty in making decisions in a short consultation (−)	
	▪patient centred +	▪miss the action in the hospital -	▪lecturers not seem to influence their perceptions (which could be because lecturers weren’t in the real world)			▪positive experience in the attachment	
	▪ the breadth rather than depth of medicine	▪it teaches skills (communication, evidence-based medicine, counselling) rather than knowledge					
	▪lacked understanding: equating general practice to part of internal medicine or a combination of all other disciplines.	▪triage patients -					
	▪private GPs more patient centred than those in the government health centres	▪lack of evidence-based practice -					
	▪relaxing posting						
Firth *et al.* 2007 [24]	▪range of case mix +	▪mundane diseases and boring -	▪peers saw primary care in a negative light: boring and for taking time off.		▪business-driven negative and stressful for some and attractive to other+/−	▪the majority of scenarios studied based within the hospital setting. This added the notion that GP was less interesting.	▪Importance of the quality and enthusiasm of the teachers to make Foundation training a success.
	▪increasing amount of medical		▪Bad speaking by hospital tutors’. It influenced perceptions			▪benefit of being taught in primary care: cases not available in hospital	
	care within primary care.		▪positive view of GP role +			▪quality of the placement was the most influential factor	
	▪“Social side” of disease (+)		▪media portrayal of the profession as major influence +/ -			▪benefits of an extended period in general Practice +	
	▪quality of care +					▪negative experiences difficult to reverse (n)	
	▪relationships +					▪the attachments improved student’s views +	
	▪multidisciplinary team +						
	▪better lifestyle but it was not an important consideration						
Mutha *et al.* 1997 [25]	▪ long –term relationship with patients vs surgical specialities that do interventions with immediate and tangible results + .	▪ the breadth of information required interfered with the ability to achieve competency and mastery -	▪clinicians (residents and attending physicians) influenced students’ career decisions +/−		▪neither debt nor future income influenced decisions.	▪perceptions developed during clinical rotations (n)	
	▪ intellectually challenging: address a variety and complexity of medical problems +		▪exposure to positive role models influenced some students’ choices +		▪Gender differences: for women, the anticipation of being in a dual-income family allowed them to minimize debt or income as a factor in their decision.	▪inpatient services tended to discount the effects of cognitive specialties.	
			▪exposure to positive role models was neither necessary nor sufficient for most of the students’ career decisions (n)				
			▪negative role models had strong dissuasive effects on specialty selections -				
			▪Women could not identify role models: deterrence from considering particular fields and created anxieties and uncertainties -				

+: Positive perceptions. -: Negative perceptions. n: Neutral perceptions.

#### Broad scope and context of practice

This extensive theme appeared in all studies and contained some linked sub-themes. All the studies indicated that medical students perceived family practice as a varied specialty with a broad practice and where the holistic perspective is necessary [16-25]. Two studies pointed out that although the medical knowledge needed for family practice is broad, it is also more superficial [20,23]. 

*“… a much more general knowledge of everything, but of a little less depth. Family physicians need much more knowledge than specialists. A family physician, if she or he has been well trained, can both remove a foreign body in an eye and treat someone suffering from a psychological problem, a depression … Something that an ophthalmologist will never take care of. That is, a family physician needs knowledge from all the specialties”*[20]*(2nd year medical student, Spain).*

The students considered that family practice allows for: continues and the long term care [16,17,19,25], to work in a community and family context [16,17,22] and to do preventive and public health activities [22] as well as home visits [17].

Five studies highlighted the special relationship between family doctors and patients [17,18,20,22,24]. In one study, students argued that private general physicians were more patient centred, than those working for government health centers [23]. This was the unique study where the investigators described a lack of understanding of primary care by the students, equating general practice to part of internal medicine or a combination of all other disciplines. 

*“To me primary care physicians . . . I’m still confused now between a primary care specialist and a physician in the hospital who is practicing as a general physician outside …”*[23]*(Final year medical student, Malaysia)*

Students believed that family practice enables flexibility and part time work, which allows having a family [16,18,19,21,22]. Nonetheless, in one study, students said that this was not an important consideration for choosing a specialty at that moment [24]. It was also noted that family practice allowed autonomy [19,21] and independence [22]. 

* “Lifestyle is important. One day I do want to be a mom, and I want to be able to spend time with my kids, and I think family is one field where you really can make your own hours. You can make your business what you want it to be, and you can do locums. You can work part-time; you can work full-time. I think that is what is so attractive about family medicine, is that you can really make a great lifestyle for yourself, outside of medicine”*[18]*(Canada).*

One study reported the perception of less medical indemnity issues compared with other specialties [16]. Other positive perceptions about family practice were the work environment, thought as friendly [22], and the advantage of working in a multidisciplinary team [24]. The management of risk and assessing urgency of undifferentiated problems were concerns reported in two studies [16,22]. Consequently, some students referred to anxiety for wanting quick answers in diagnosis [22]. 

*“In general practice, I just felt that sometimes they were over-investigating and sometimes under-investigating… I didn’t feel I could tell sufficiently who I wanted to investigate… I just found that particularly scary”*[22]*(Final year medical student, United Kingdom).*

The issue of rural family practice emerged in two studies. In general, students thought it was a heavy workload, with long working hours and a lot of responsibility [16,19]. Some other students thought there should be a compulsory rural term [19].

#### Lower interest or intellectually less challenging

All the studies reported that medical students perceived family practice as not intellectually challenging [19,20] because it treats common diseases [17,23] and serious problems are referred to specialists [16,21,22]. It was also regarded as superficial, “mundane” and repetitive [20,22-24]. 

*“I’d never really go to my general physician other than mundane things, well they seemed to be mundane for me . . . it often seems that the good bits were taken by other places and the general physician was the person who saw the coughs and colds”*[24]*(United Kingdom).*

It was pointed out that there was less action and less technology than in hospitals [22,23]. Some students thought that family physicians are the gatekeepers of the health care system [20] and that they just triage patients [23]. In one study, students argued that choosing family medicine seems to limit oneself, especially for high-achieving students [18]. The idea of a quasi-administrative medical practice emerged in some studies [16,20,22] and also the idea that family physicians suffered from lack of time and professional isolation [19,22]. 

*“I know that… most specialties, the amount of time you can spend with a patient is restrictive, but I felt particularly in general practice often that the time really was limited and you often couldn’t spend as long with a patient as the patient really needed or you wanted to spend with them”*[19]*(Australia).*

One study reported that family practice teaches skills like communication and counselling rather than knowledge and that the students felt a lack of evidence based practice [23]. Only one study reported that family practice was intellectually challenging as it addresses both variety and complexity of medical problems, but clarifying that the breadth of information required can interfere with the achievement of competency and mastery [25].

#### Influence of role models and society

Negative comments and attitudes from other specialists, teachers, residents, colleagues and peers about family practice had an influence on students’ career interests [16,18-21,24,25]. In one study, students pointed out that derogatory comments had no influence on their career choices [22]. Another study said that negative opinions from lecturers do not seem to influence on students perceptions and that it is so because students perceived lecturers to pertain to the academic world, not to the “real world” [23]. 

*“I’ve also found with specialists, I think they’re pretty hard on GPs as well every specialty lecture they give, oh bloody GP did this, sort of thing”*[16]*(Australia).*

Several studies reflected that students felt pressure from family, friends and society to choose a different specialty [16,20,21]. 

*“There’s still quite a stigma attached to it and I know this shouldn’t affect me, but everyone I meet, it’s like “You’re not going to be a GP are you? You haven’t worked so hard to be a GP”. It’s almost like it’s not a proper doctor”*[21]*(final year medical student, United Kingdom).*

*“Now people think that you will finish your studies and you will not be a family physician: you have to be a neurosurgeon and, if possible, in Barcelona… Less than that, you have spent six years of your life, and you have thrown them to the garbage”*[20] (2^nd^*year medical student, Spain).*

The influence of role models on students’ perception, either positive or negative was identified in five studies [17-19,24,25]. In one study, participants said that exposure to positive role models was neither necessary nor sufficient for their career decisions [25]. This study was the only to identified a gender difference: women could not identify role models and this was a deterrence from considering particular fields and created anxieties and uncertainties [25]. 

*“The problem was that when I went through ob-gyn (here), there were really no women attending; there was one that wasn’t really impressive or that I would aspire to be like, so I think that was one of the problems I had deciding to go into obstetrics and gynaecology”*[25]*(woman, 26 years old, USA).*

The negative media coverage of family medicine was also identified as an important factor on students’ perception in three studies [19,21,24]. 

*“I like it how on GP dramas and things on television they always seem to have the time to go for lunch and sit and chat to their spouses and things when they’re out for lunch”*[24]*(United Kingdom).*

#### Lower prestige

Five studies reported students’ perception of lower status of family medicine compared to other specialties, either professionally (being at the bottom of the medical hierarchy), or socially (decreasing its social role) [18-22]. One of the studies stated that the lower status was not always an influence on students’ career decisions [22]. 

*“There is a very clear hierarchy in medicine, and family medicine is at the bottom… Above lab but below medical specialties. Surgery has always had much prestige”*[20]*(6*^th^*year medical student, Spain).*

*“Only the fact that the family physician works in a community health centre, and one can go there for everything, it looks like family medicine practice is of less importance… People think that, when you finish your undergraduate studies, you can practice as a family physician; they are not aware it is a medical specialty”*[20]*(2*^nd^*year medical student, Spain).*

*“family physicians in Spain are undervalued, but they play an important role in other countries… Here, they have no authority. From this everything goes down because they do not have the social prestige they used to have…”*[20]*(2*^nd^*year medical student, Spain).*

Some students considered the choice of family medicine as an inferior choice, a second choice residency, being a necessary specialty but undesirable as a career option [18-20]. Two studies reported that family medicine was considered as a second career that follows working first in a subspecialty [17,22]. 

*“I do see general practice as, as maybe an option once I’ve pursued the surgical route”*[21]*(final year medical student, United Kingdom).*

*“Well… (pause) as for my thoughts right now, I am leaning towards emergency medicine after graduation… I want to achieve a sufficient level of competency, then, for example ten years later, when it becomes physically burdensome, well, I think I will want to go into primary care”*[17]*(Japan).*

#### Low remuneration

The theme of low remuneration was discussed in six studies. Three of them mentioned the poor remuneration compared to other specialties as a reason not to choose family medicine [16,18,20], and the difficulty to generate an additional income in the private sector [20]. 

*“That was a big factor, actually. That was really stressful in terms of that factor making a decision because you see the amount of debt you’re in, or the amount that I was in, or am in, from medical school and my previous education”*[18]*(Canada).*

On the other hand, Edgcumbe *et al*[22] reported that students thought that General Practitioners^a^ were well paid or overpaid, particularly at earlier stages of their careers. Mutha *et al*[25] reported that neither debt nor future income influenced students’ decision. This was again the mere study that identified a gender difference: for women, the anticipation of being in a dual income family allowed them to minimize debt or income as a factor in their decisions.

The business aspects of running a practice were perceived in a negative light and stressful for some students [22,24] while it was a positive factor for others [24]. In one study carried out in the UK, students thought that the 2003 GP contract impinges on the professional autonomy [22]. 

*“With these stupid government targets, everyone who comes through the door who’s got hypertension has to have this, has to have that, and you try to accumulate points which I think takes away a little bit of your clinical own judgement”*[22]*(Final year medical student, United Kingdom).*

#### Medical School influences on Specialty Choice

Students felt that undergraduate experiences in GP were significant and influenced in their career intentions [16,20-22,25]. Some said that the exposure was more stimulating than expected because it needed hands-on experience and no just observation [19], while others perceived early experience as not real medicine [21], and some others reported a disparity between training and practice [23]. 

*“I don’t really agree with what we were taught. We were taught you need to listen to the patient, take the history as well as counsel them. So, all in all, definitely things will move on at least 10–15 minutes. Judging from the amount of patients that come to primary care, that’s why you see some of the doctors tend to skip through . . . . They just speak a few words, not even sentences. Even when the patient wants to ask anything, they just say, “OK, OK, next!” I mean the impression they give me wasn’t that good*” [23]*(Final year medical student, Malaysia).*

*“I thought GP world be pretty boring… but to the honest, it (the GP attachment) opened my eyes quite a lot in that I saw lots of interesting cases, and you don’t really know what’s going to come through the door next*[22]*(Final year medical student, United Kingdom).*

One study reported almost no exposure to family medicine practice so the students had poor idea of what family medicine practice was [19]. The length and quality of the exposure and also the atmosphere during the practices were important elements that may influence the specialty choice [17,24]. Some students thought that there was little representation of GP in the curriculum and that medical education was still mainly hospital based [18,19,24]. One study reported the benefit of being taught in primary care, learning from cases not available in the hospital [24]. 

*“Just from other students, it seems to be the people who’ve had some really good GPs as supervisors, they’re keen to do general practice”*[16]*(Australia).*

*“The worst part is we don’t have any exposure to family physicians until third year”*[18]*(Canada).*

*“…maybe this is one of the reasons why we are not attracted by family medicine: because actually we do not know what family medicine is. We finish our undergraduate studies and think that family medicine practice mostly consist of signing drug prescriptions, but this practice might have another content nobody has taught to us”*[20]*(6*^th^*year medical student, Spain).*

#### Post graduate training

This theme emerged in five studies: the idea of a less intensive and shorter training was discussed in two studies and was considered as a positive element [16,18]. The flexibility, well-structured programme and the lack of competition compared to hospital training were also positive aspects of family practice training [22]. In one study it was noted that the lack of research in the training was considered as either a positive or negative aspect depending on the students [22]. The Spanish study reported that students thought that the fourth year residency programme was unnecessary [20].

### Synthesis

After identifying these seven key themes, were also looked into patterns in the distribution of these themes among studies. This was conducted by comparing and contrasting the themes against the country in which the study took place, the phenomenon of interest, the method of data collection (focus groups or single interviews), the method of analysis and the characteristics of the study population (sex, age and year of medical course) and the type of university (public or private). No systematic pattern connected to any of these factors or any other was found.

## Discussion

### Summary of Main Findings

Our qualitative review provides a comprehensive picture of medical students’ attitudes towards family medicine in the available literature. In general, although some students find family medicine appealing, it is regarded as a career of low interest and prestige.

This review moreover shows that medical students know some of the most important characteristics and aspects of the scope of practice in family medicine. The most repeated positive aspects being the continuity of care, the holistic approach and the relationship with patients. The idea that family medicine allows for flexibility and a good lifestyle which facilitates having a family was also repeated in most studies.

Generally, students had the perception that family medicine is a specialty with lower interest and intellectually less challenging than other specialties. Role models, either positive or negative, were identified as important factors that influence their perceptions. The negative attitudes from other specialists, teachers and peers also seem to play an important role, as well as media coverage. Additionally, the perception of lower prestige, and sometimes a salary lower than in other specialties was reported in many studies.

Medical school curricula and exposure to family practice was an important factor on specialty choice. It should be noted that some students expressed a change in their perceptions (towards positive) after exposure to family medicine [22,24]. Some positive thoughts about aspects of postgraduate family medicine training were the short duration, the lower intensity of training and the work environment. Nevertheless, two studies reported negative views about this theme: the ease of matching with family medicine [18] and, in the case of the Spanish context, the long duration of the residency programme (4 years) [20].

### Strengths and limitations of the study

One of the strengths of this study is that an extensive effort was made to find all relevant primary studies by performing an exhaustive bibliographic research in six different databases. Additional information was obtained from eight of the ten authors from the included studies. Nevertheless, it cannot be excluded that there might be studies published in other languages other than English, Spanish, Italian, Portuguese or French. Seven of the ten studies selected were conducted in Anglo-Saxon countries, of which the differences among all of them in terms of academic, health care and societal contexts are large. Notwithstanding, the performed exercise of identifying the most prevalent and convergent students’ perceptions of family medicine across countries further increases the confidence in our results. Finally, the originality of the work lies in the fact that, to our knowledge, there is no other qualitative synthesis available on this topic.

Our study has some limitations. While the goal of this review was to investigate medical students’ attitudes and perceptions about primary care and family medicine, half of the included studies focused primarily on identifying factors that influence a career interest in medical students. Although the students expressed their own views about family practice it is plausible that other themes may have emerged if these studies had focused specifically on their perceptions and attitudes. Another inherent limitation of performing a synthesis is that the confidence in its results depends partially on the quality of the included studies. In our synthesis the quality of the included studies was generally high. On the other hand, despite efforts to find common patterns among the themes identified, it was only managed to extract and combine the results, providing a lower interpretative level than if we had been able to undertake a metha-synthesis had been undertaken.

Most primary studies were carried out by investigators related to family medicine. This could introduce some bias in favour to better attitudes and perceptions towards family medicine than if the investigators have been from other medical specialties. Nevertheless, as our results are in general negative, this possible bias have no changed the direction of our findings, although it may have diluted the expression of some worse perceptions.

### Comparison with existing literature

The majority of the literature related to this area focuses on the study of the factors that influence students to choose a medical specialty. A study with graduate students that joined a family medicine residency programme identified some perceptions similar to those identified with medical students [26] (scope of practice, diversity of the work, freedom to shape practice to best meet individual and community needs, and presence of family medicine role models). A previous literature review about factors related to the choice of family medicine also found that faculty role models were related to specialty choice, serving as both positive and negative [7].

Students’ perception of poor remuneration of family practice has also been reported in quantitative studies. For instance, a survey of 781 medical students in the University of Toronto about their perceptions of physician remuneration showed that between 85% and 89% of students perceived that family physicians were paid too little [9]. However, in two prior narrative reviews, there was not a clear-cut relationship between debt and specialty choice [7,27]. One of these reviews was about factors related to the choice of family medicine and identified the lack of prestige, low income potential and low intellectual content of the specialty as factors concerning students rejecting family medicine [7].

It has already been described that experiences at medical school are strong determinants of attitudes towards the medical specialties. Consequently, attitudes are the most important factor that determines a specialty choice [28,29]. As in our work, two systematic reviews [7,8] state that medical school experiences are an important factor related to the choice of primary care.

Finally, a systematic review identified the influence of medical school exposure to family practice and the culture of the institution as factors associated with medical students’ choice of a primary care specialty [27]. In our review, one study claimed the benefit of an earlier exposure to family practice, although one of these reviews found no evidence that inclusion of family medicine courses in first and second year curricula was related to the choice of family medicine. However, required family medicine time in the third or fourth year was positively related to higher numbers of students selecting family medicine [7].

### Implications of our results and future research

The findings of our qualitative review improve our understanding of medical students’ perceptions and attitudes towards primary care and family medicine across countries. Through the identification of seven overarching themes, the most important contribution of our study has been to emphasize on the main convergent of medical students’ perceptions and attitudes towards family practice despite contextual differences among the different studies here considered.

Furthermore, this work may help identifying interventions that could be applied in medical schools in order to create an impartial academic atmosphere including: 1) one with a larger representation of family medicine in the curricula; 2) an increase in the ratio of lecturers related to family practice; 3) a scheduled undergraduate exposures in family medicine considering duration and quality; and 4) more positive attitudes towards family medicine on the part of lecturers, specialists and residents. In order to successfully implement these strategies, they should be embedded within structured study programmes.

As the students’ perception towards one specialty is a crucial factor in the process of choosing their career preferences, it should be important to study some of the identified factors in more detail. Our research does not only suggest some interventions at the academic level but also in a broader context. In this sense, it could be interesting to explore the relationship between remuneration and prestige within family medicine.

## Conclusions

Our qualitative review provides a comprehensive picture of medical students’ attitudes towards family practice. The available evidence shows that in general, family medicine is regarded as a career of lower interest and prestige. Role models, negative attitudes from others, medical school curricula and exposure to family practice seem to play an important role in this perception.

## Endnotes

^a^Family physicians and general practitioners are equivalent and correspond to different national ways to designate the specialty.

## Competing interests

The author(s) declare that they have no competing interests.

## Authors’ contributions

PAC, AMZ and JJM conceived the study. AS performed the search strategy. AS and GMD selected the included articles, extracted and analysed data. AS and PAC wrote the first draft. All authors reviewed the manuscript critically for important intellectual content and have given final approval of the version to be published.

## Authors’ information

UNIVERSIDAD Y MEDICINA DE FAMILIA (UNIMEDFAM) Research Group

Amando Martín Zurro, Josep Jiménez Villa, Antonio Monreal Hijar, Xavier Mundet Tuduri, Ángel Otero Puime, Pablo Alonso-Coello, Cristina Aguado Taberné, Pablo Bonal Pitz, Francisco Buitrago Ramírez, Concepción Carratalá Munera, Verónica Casado, Vicente, María Teresa Delgado Marroquin, Ramón Descarrega Queralt, Manuel Gálvez Ibáñez, Antonio J. García Ruiz, Luís García Olmos, Vicente Francisco Gil Guillén, José Manuel Iglesias Sanmartin, Carmelo Jiménez Mena, Emilio Lara Valdivieso, Inés Lizaga Castillón, Antonio de Lorenzo-Cáceres Ascanio, Jorge Martínez de la Iglesia, Flora Martínez Pecino, Juan Francisco Menárguez Puche, M. Pilar Navarrete Durán, Jorge Navarro Pérez, Domingo Orozco Beltran, Eduard Peñascal Pujol, José Ramón Rodríguez Borges, Pilar Rodríguez Ledo, Juan Alfonso Romero Furones, Mercedes Sánchez Martínez, Juana Agustina Santana Caballero, Orlando Segura Álamo, Antonio Solbes Caro, José Zarco Montejo.

## Pre-publication history

The pre-publication history for this paper can be accessed here:

http://www.biomedcentral.com/1472-6920/12/81/prepub

## Supplementary Material

Additional file 1 Search Strategy. Click here for file

Additional file 2 Summary tables of included studies. Click here for file
