# Supplementary material for: Medical students’ perceptions and attitudes about family practice: a qualitative research synthesis
Source: BMC Med Educ. 2012 Aug 21;12:81. doi: 10.1186/1472-6920-12-81 (PMC3546071; doi:10.1186/1472-6920-12-81)
Supplement: Additional file 1 — Search Strategy. [file 1472-6920-12-81-S1.docx]

**Additional file 1. Search Strategy**

We searched PubMed, EMBASE, CINAHL, SCI-Expanded, SSCI, ProQuest Dissertations & Theses, from their inception dates until July 2010.

- **Medline search strategy:** 1131

(Accessed via PubMed, 05/July/ 2010)

#1 Students, Medical"[Mesh] OR student*[tiab].134286

#2 attitude[Mesh] OR perceptions[Mesh] OR attitude*[tiab] OR perception*[tiab] OR career choice [Mesh] OR choos* [tiab] OR choice* [tiab]. [745181](http://www.ncbi.nlm.nih.gov/pubmed/advanced?cmd=HistorySearch&querykey=6&)

#3. Search Primary health care [Mesh] OR Physicians, Family [Mesh] OR Family Practice [Mesh] OR primary care[tiab] OR primary healthcare[tiab] OR primary health care[tiab] OR family practice[tiab]. 148152

#4 ((#1) AND #2) AND #3.1841

#5 cross sectional [tiab] OR survey[tiab] OR surveys[tiab] OR questionnaire*[tiab]50426

#6 (#4) NOT #5 .1131

- **EMBASE search strategy:** 417

Database: EMBASE <1980 to 2010 Week 27>

1 exp medical student/ (20595)

2 student*.ti,ab. (72990)

3 1 or 2 (80596)

4 attitude/ (24149)

5 exp perception/ (105080)

6 (attitude* or perception*).mp. (204067)

7 exp primary health care/ (49534)

8 exp general practitioner/ (33688)

9 exp general practice/ (24977)

10 (primary care or primary health care or primary healthcare or family practice).mp. (54741)

11 4 or 5 or 6 (258743)

12 7 or 8 or 9 or 10 (106670)

13 3 and 11 and 12 (758)

14 (cross sectional or survey or surveys or questionnaire*).ti,ab. (391179)

15 13 not 14 (417)

- **CINAHL search strategy:** 374

(accessed via EBSCO, July 2010)

S1 (MH "Students, Medical") (3486)

S2 TI student* or AB student*(51588)

S3 (MH "Attitude+")(149133)

S4 (MH "Perception+")(22051)

S5 TI attitude* OR AB attitude* OR TI perception* OR AB perception* (55958)

S6 TI choice* OR AB choice*(23308)

S7 (MH "Primary Health Care") (21009)

S8 (MH "Physicians, Family") (5965)

S9 (MH "Family Practice") (7521)

S10 TI primary care OR AB primary care OR TI primary healthcare OR AB primary healthcare OR TI primary health care OR AB primary health care OR TI family practice OR AB family practice (25068)

S11 S1 OR S2 (52880)

S12 S3 OR S4 OR S5 OR S6 (208224)

S13 S7 or S8 or S9 or S10 (43476)

S14 S11 OR S12 OR S13 (374)

- **Science Citation Index Expanded (SCI-EXPANDED) -**1899-present

**Social Sciences Citation Index (SSCI) -**1956-present

(accessed via ISI Web of Knowledge) 13/.July./2010: **398**

# 1 >100,000 Topic=(student*)

# 2 >100,000 Topic=(attitude* OR perception*)

# 3 >100,000 Topic=(primary care OR primary health care OR primary healthcare OR family practice)

# 4 834 #3 AND #2 AND #1

# 5 >100,000 Topic=(cross sectional OR survey OR surveys OR questionnaire*)

# 6 398 #4 NOT #5

- **ProQuest Dissertations & theses: 48**

(2004 –07/June/ 2011)

((student*) AND (attitude* OR perception*) AND (primary care OR primary health care OR primary healthcare OR family practice)) AND NOT ((cross sectional OR survey OR surveys OR questionnaire*))
