# Supplementary material for: Medical students’ perceptions and attitudes about family practice: a qualitative research synthesis
Source: BMC Med Educ. 2012 Aug 21;12:81. doi: 10.1186/1472-6920-12-81 (PMC3546071; doi:10.1186/1472-6920-12-81)
Supplement: Additional file 2 — Summary tables of included studies. [file 1472-6920-12-81-S2.docx]

**Additional file 2. Summary tables of included studies**

1. Tolhurst H, Stewart M. Becoming a GP. **A qualitative study of the career interest of medical students**. Australian Family Physician. 2005;34:204-6.
2. Saigal P, Takemura Y, Nishiue T, Fetters MD. **Factors considered by medical students when formulating their specialty preferences in Japan: findings from a qualitative study.** BMC Medical Education. 2007;7:31
3. Scott I, Wright B, Brenneis F, Brett-MacLean P, McCaffrey L. **Qualitative studies review about attitudes and perceptions of medicine students about family practice and primary attention**. Can Fam Physician. 2007; 53: 1956-7.
4. Thistlethwaite J, Kidd MR, Leeder S. **Enhancing the choice of general practice as a career**. Aus Fam Physician. 2008; 37: 964-8.
5. López-Roig S, Pastor MA, Rodríguez C. **Understanding the reputation of and professional identification with the practice of family medicine among medical students: a Spanish case study.**
6. Hogg R, Springgs B, Cook V. **Do medical students want a career in general practice? A rich mix of influences!** Education for primary care.2008;19:54-64.
7. Edgcumbe DP, Lillicrap MS, Benson JA. **A qualitative study of medical students’ attitudes to careers in general practice.** Educ Prim Care. 2008; 19: 65-73.
8. Chirk-Jenn NG, Kwok-Chi L, Cheong-Lieng T. **What do medical students think about primary care in Malaysia? A qualitative study**. Edu Prim Care.2005;16: 575-80.
9. Firth A, Wass V. **Medical students’ perceptions of primary care: the influence of tutors, peers and the curriculum.** Edu Prim Care.2007;18:364-72.
10. Mutha S, Takayama JI,O’Neil EH. **Insights into medical students’ career choices based on third-and fourth-year students’ focus-group discussions.** Acad Med. 1997; 72: 635-40.

1.Tolhurst H, Stewart M. Becoming a GP. **A qualitative study of the career interest of medical students**. Australian Family Physician. 2005; 34:204-6.

| **Author, year, country** | **Objective** | **Study design** | **Participants** | **Quality** | **Commentaries** |
| --- | --- | --- | --- | --- | --- |
| Tolhurst 2005  Australia | Identify and understand factors influencing career interest of medical students. | Qualitative methodology.  **Data collection:**  10 focus groups of 6-10 medical students.  **Analysis:**  Sessions taped, transcribed and analysed for emergent themes using computer software.  Two researchers coded and checked independently the data (triangulation) and discussed their results to come to a consensus on the meaning of the data.  Data was analyzed thematically.  Data reached saturation. | 1rt or final year medical students from 3 universities and a national student conference on rural health.  82 students (36 men, 46 women). Age range: 18-40.  Rural and urban background.  Some were aboriginal, non-English speaking, single, married, with and without children, graduate students with years of experience in the workforce and students who entered medicine as school leavers. | High | **Conclusions:**  -Undergraduate experience is an important influence on medical students’ career choices.  -Some reasons for students not are attracted to general practice: it is poorly paid, work long hours with a large burden of responsibility.  -Specialists denigrating general practitioners and the low status of general practice in the community influenced some students. |
| **RESULTS: EMERGED THEMES** | | | | | |
| **First year students:**  -General practice (GP) offers diversity, variety and continuity of care.  -GP allows working in a community and family context.  -GP offers an opportunity to use pre-existing skills.  -Rural practice gives the opportunity to practice a wide range of skills (procedural and hospital care).  -Many women saw GP as providing flexibility with their plans for having a family, availability of part time and flexible training, part time work after graduation and the portability of GP qualifications.  -Discomfort with the inherent uncertainty of GP such as assessing the urgency of undifferentiated problems.  -Work of urban general practitioners is limited with any serious problems being referred to specialists.  -Poor remuneration.  -Rural general practice is associated with workload, long work hours, on-call commitments and levels of responsibility.  -Amount of paperwork and professional isolation.  -Less medical indemnity issues compared with other specialties, less intensity and length of training, less long working hours and no teaching hospital culture.  **Last year students:**  -Their undergraduate experiences had influenced their career preferences.  -GP’s negative attitudes were related with remuneration, workload, red tape and medical politics.  -Specialists and teachers had negative attitudes to GP.  -Some felt that family and friends would be disappointed if they became general physician instead of entering specialist practice | | | | | |

2.Saigal P, Takemura Y, Nishiue T, Fetters MD. **Factors considered by medical students when formulating their specialty preferences in Japan: findings from a qualitative study.** BMC Medical Education. 2007;7:31

| **Author, year, country** | **Objective** | **Study design** | **Participants** | **Quality** | **Commentaries** |
| --- | --- | --- | --- | --- | --- |
| Saigal-2007  Japan | -Identify factors considered by Japanese medical students when formulating a preference to pursue a primary care versus subspecialty care career.  -Examine the understanding of family medicine, primary care and subspecialty practice. | Qualitative methodology.  **Data collection:**  -Semi-structured interviews (39 min.) to medical students. Digitally audio taped.  -Informal interview with academic faculty.  -Field notes.  **Analysis:**  Interviews were transcribed by one author. Two authors did the primary analysis and identified themes by multiple readings of transcripts.  Development of a conceptual model depicting the pathways to a career in primary care in Japan and another in the U.S.  Member checking by discussing the primary findings with some of the students interviewed.  To validate findings the authors had in-depth discussions with Japanese and American physicians who have expertise in the heath care systems. | Medical students from 3^rd^ to 6^th^ years course at Mie University Medical School.  N=25 (17men, 8 female).  Age: 21-35 years  8 preclinical and 17 clinical students.  Half of the participants preferred primary care  specialization at the time of the study. | High | **Conclusions:**  -Preclinical and clinical experiences and role models are influential factors on the formulation of specialty preferences.  -Most students have at best, a rudimentary understanding of family medicine as a discipline.  **Limitations:**  -The host Department at Mie University is leader in the establishment of family medicine in Japan and students volunteering for the study may have known the departmental bias towards primary care.  -Small sample size (about 6% of eligible 3^rd^ to 6^th^ year students). |

| **RESULTS** |
| --- |
| Motivations for entering the medical profession:  -Personal experiences with illness or witnessing their loved ones suffer from illness influenced their interest in medicine and often their specialty choice.  Factors that affected their specialty preference:  -Academic experience during preclinical training, lab research, basic science course work, or extracurricular activity affected their specialty choice.  -Clinical experience during clinical rotations.  -Opportunity to have direct patient contact, length of clinical exposure, quality of interactions with faculty physicians, personality of physicians, presence of a physician role model or mentor and the overall atmosphere within departments.  -Students’ views on primary care, subspecialty care and family medicine.  -Primary care is seen as a second career in medicine that follows an initial stepping-stone of 10-15 years of training and working as a sub specialist.  -Views regarding primary care physicians:  *Community based  *Primary consultation before seeing specialists  *Common disease, easy to treat.  *Psychosocial concerns  *Long term care  *Good doctor-patient relationship  *Treat the whole body.  *Broad knowledge than specialist care.  *Care for entire family  *Focus on prevention, triage and medical interviews.  *Home visits are important  *Prevention of disease in the community |

3.Scott I, Wright B, Brenneis F, Brett-MacLean P, McCaffrey L. **Qualitative studies review about attitudes and perceptions of medicine students about family practice and primary attention**. Can Fam Physician. 2007; 53: 1956-7.

| **Author, year, country** | **Objective** | **Study design** | **Participants** | | **Quality** | **Commentaries** |
| --- | --- | --- | --- | --- | --- | --- |
| Scott -2007. Canada | Describe the factors that medical students report influences them to pursue careers in family medicine (FM). | Qualitative methodology: phenomenology.  Purposeful sampling  **Data collection:**  -26 focus groups (24 students)  -9 individual interviews  **Analysis:** Two investigators read each set of transcripts independently. Thematic content analysis. Triangulation, member checking | 33 medical students from 3 different universities.  27 women/ 6 men; Distribution of participants in each university: 14 /10/9  End-of-preclinical data: 18 students (2003).  End-of-clinical data: 15 students (2006). | | high | **Strengths:** Purposeful sampling to identify 2 groups of students (those who maintained a career interest in FM and those who changed their interest to FM) at 3 medical schools with different cultures and curriculum structures. A small number of students who were not interested in FM medicine or who had switched from FM were included and their comments confirmed the comments made by the bulk of respondents who were interested in family medicine.  **Limitations:**  -Overrepresentation of women  -Participants in the focus groups were primarily those interested in family medicine and were not representative of all undergraduate medical students. |
| **RESULTS: EMERGED THEMES** | | | | **CONCLUSIONS** | | |
| -Pre-medical school influences: role models, positive and negative, were the most important influences on career interest.  -Medical school influences:  -Little representation of FM in the curriculum.  -FM often appeared in a negative light by other specialists  -Role models (positive and negative) are important influences.  -The broad scope of practice prompted students to consider FM as an option.  -Postgraduate training influences:  -The shorter and physically less demanding FM residency.  -The culture of the FM residency was appealing  -Choosing FM is characterized by students as limiting oneself, particularly for high-achieving students.  -The concept of FM as a backup or second-choice residency.  -The lower prestige associated with FP residence.  -Difficulty in retraining later in their careers if they choose FM.  -Life-in-medicine influences  -FM is associated with good lifestyle and flexibility  -The scope of practice, particularly in rural settings, and enduring relationships with patients  -Worries about prestige and income during their practice life. | | | | -Medical schools could preferentially select students who report an interest in FM at medical school entry.  -Medical educators should increase the presence of family physicians in the preclinical years, as medical students perceive an inequity in this area.  -Medical schools and departments of FM should try to reduce the negative characterizations of FM by both other specialists and family physicians themselves in the preclinical and clinical years.  -Students should receive high-quality FM clinical experiences with positive role models to improve their perceptions of careers in FM.  -Students should be provided with accurate information on what the training and future life expectations are for residencies and careers in family medicine.  -Future research should investigate what information and what kinds of experiences are particularly valuable in aiding medical students in their career choices. | | |

4.Thistlethwaite J, Kidd MR, Leeder S. **Enhancing the choice of general practice as a career**. Aus Fam Physician. 2008; 37: 964-8.

| **Author, year, country** | **Objective** | **Study design** | **Participants** | | **Quality** | **Commentaries** |
| --- | --- | --- | --- | --- | --- | --- |
| Thistlethwaite2008  Australia | -Explore factors that influence students and junior doctors to choose or reject a general practice (GP) career.  -Triangulate the findings of a literature review with the views of a selection of stakeholders. | Qualitative methodology: phenomenology  **Data collection:** Semi structured phone interviews, audio taped and transcribed. Interview questions were based on the literature findings to explore their correlation with the views of a sample of Australian students and doctors.  The questions were not piloted but were adapted and refined in each interview.  **Analysis:** Thematic analysis. | -13 medical students (3 male, 10 women)  -5 junior doctors (2 male)  -5 general practice registrars (3 male)  -15 GPs (8 male) | | High | The views of the stakeholders were similar to the conclusions drawn from the 198 papers reviewed.  **LIMITATIONS:** The sample of interviewees is not truly representative |
| **RESULTS: EMERGED THEMES** | | | | | | |
| 1.Factors affecting career choice in GP:  -Medical education still mainly hospital based.  -GP seen as inferior choice  -Role models  2. GP exposure at medical school and for junior doctors:  -Comparison of GP with hospital.  -Effect of GP attachments.  -Generalist versus specialist.  -Having GP exposure earlier during training.  -More stimulating than expected.  -Needs hands-on experience not just observation.  -Perceptions of GP while a student.  -Sell FP as a great job.  -Prevocational GP Placements Program.  3. There are negative views of GP expressed during hospital terms by hospital doctors without no reasons.  4.Attractions of GP as a career:  Continuity of care, flexible working hours, career path, lifestyle, stimulating and lots of variety, working with people, autonomy, prestige, skill mix, social status, holistic care.  5.Making GP more attractive:  Less bureaucracy, increase flexibility, reduce government interference, better communication between GPs, pay, portray as enjoyable career, doing procedures, enhanced recognition of GPs, more support, less time pressure, students to gain better understanding of role, increase availability of part time training. | | | | 6.What makes GP unattractive:  -Lack of support.  -Lack of time with patients.  -Decreasing prestige.  -It is not seen as a specialty or as intellectually challenging.  -Negative role models and negative media coverage.  7. GP teaching  -Enjoyable to have students in practice  -General practitioners not trained to teach.  -Remuneration factors.  -Increases status.  8. Teamwork and effects on choice  -Attractions of interprofessional teams.  -Lack of training in teamwork.  -Team as support.  -Teamwork important.  9. Effects of rural attachments.  -Compulsory rural term.  -Reasons for being rural GP.  -Hard work. | | |

5.López-Roig S, Pastor MA, Rodríguez C. **Understanding the reputation of and professional identification with the practice of family medicine among medical students: a Spanish case study.**

| **Author, year, country** | **Objective** | **Study design** | | **Participants** | **Quality** | **Commentaries** |
| --- | --- | --- | --- | --- | --- | --- |
| López-Roig 2010  Spain.  This investigation integrates a larger international project | Explore the reputation of (thoughts upon the family medicine specialty) and professional identification processes (pros and cons of pursuing a career as a family physician) with family medicine practice among medical students. | Qualitative case based research.  **Data collection:**  -6 focus groups of 6-9 students (3 meetings per group). Recorded and transcribed verbatim.  -Complementary information by documentary analysis of a variety of documents (academic material, health ministry reports).  **Analysis:** Discursive thematic analysis.  Initial inductive coding using the software package N-Vivo 7.0 was done separately by two authors. The initial list of codes was discussed among the 3 authors iteratively until reach a definitive set of themes.  -Verification strategies: methodological coherence. | | 48 medical students:  27 in 2^nd^ year and  21 in 6^th^ year of a medical faculty. | High |  |
| **RESULTS: EMERGED THEMES** | | | | | | |
| 1. The practice of Family Medicine (FM).  *FM as focused on human patient-physician relationship *vs* technology.  *Family practice as superficial, repetitive and with lack of intellectual challenge. A quasi-administrative medical practice.  *Family physicians’ clientele is elderly.  *Broad practice.  *Family physicians’ lost of social role  2. FM knowledge and skills.  *Family physician as the kindest and more tolerant doctor.  *FM, the largest breath but depthless medical wisdom.  *6th year medical students perceive the 4-year residency program in FM is unnecessary.  *The role of family physician in the health care system is gatekeeper and care coordinator. First medical contact and referrer to specialties.  3. Family medicine prestige  *FM at the bottom of the medical hierarchy.  *Unknown status of FM as a medical specialty. | | | *The low status is associated with lower salaries, less impressive facilities and devalued type of knowledge needed to practice.  *Population and health care decision-makers do not appreciate FM because of the high volume of administrative tasks and the accessibility outside the hospital and the belief that it is not a specialty.  *The low professional prestige is concomitant with a lack of clear medical identity (6th year student). The lack of professional recognition is related with the limitations of FM practice in the Spanish context (2nd year students).  4. Attitudes vis-à-vis FM as a career choice.  *FM as necessary for the health care system, but undesirable as a career option.  *Undergraduate studies experiences as significant for career choice.  *Students only consider quality of life when choosing specialties other than FM.  *Unattractive to students because of the lower probability of generating additional income when practicing in the private sector.  *Social and academic persuasion for not choosing FM. | | | |

6.Hogg R, Springgs B, Cook V. **Do medical students want a career in general practice? A rich mix of influences!** Education for primary care.2008;19:54-64.

| **Author, year, country** | **Objective** | **Study design** | **Participants** | **Quality** | **Commentaries** |
| --- | --- | --- | --- | --- | --- |
| Hogg-2008  UK | Explore in depth the factors which may influence final year medical students in their intentions regarding a career in General Practice (GP). | Combination of quantitative and qualitative methods.  **Data collection:**  -2 fixed choice questionnaires: before medical school and after the final year general practice attachment.  -3 focus groups: 5-7 students after the final year practice module.  Topic guide influenced by the main themes from questionnaire responses.  The focus group started with a brain-storm using photos of well-known hospital doctors and GPs from the media.  Focus group were recorded and transcribed.  **Analysis:** using the Framework method described by Ritchie and Spencer.  An independent qualitative analysis was carried out to ensure rigour.  Discussion was held and agreement reached between the two analyses. The process was overseen by a third researcher. | Convenience sampling.  30 final year medical students passing through their 4 week final year general practice module (FYGPM) in January, February and March.  Inclusion criteria: students attended the briefing seminar on the first day of the module. | High | **LIMITATIONS**  -The proximity of the FYGPM placement may have introduced bias. Repeating the study after an interval could reduce this.  -Small sample size. A fourth focus group could have been used to formally test the emerging theories, though informal testing was carried out in the second and third focus groups with some adjustment to topics discussed based on findings from previous groups. |

| **QUALITATIVE RESULTS: EMERGED THEMES** | **QUANTITATIVE RESULTS** |
| --- | --- |
| 15 students took part in the focus groups.  1. Negative perceptions of GP as a career.  -Lower status than hospital-based careers.  -Bad mouthing from family.  -No attractive media role models.  -Bad mouthing by hospital doctors about their GP colleagues.  -Perception of the early experiences as not “real” medicine.  2. Positive perceptions of GP as a career.  -The FYGPM led to a shift in students’ perceptions of a GP career.  -Benefits of flexible working.  -Varied and challenging  3. The experiencing reward is difficult to reach for a student when he is in a GP attachment because the time is very short.  4.Control:  -GPs were perceived to work outside the medical hierarchy so they have more control over their financial affairs, working hours and lifestyle.  -GPs have lower lever of control over the medical care of their patient and have to refer to specialist.  5. Many students chose to keep GP in reserve, as a back-up career when they wish to make their life external to medicine a priority.  6. Perception of GP with a special interest as a good alternative as they have the best of both worlds. | Response rate of questionnaires pre-FYGPM: 95,8%.  Response rate of questionnaires post-FYGPM: 72,3%.  Intentions:  -The number of students considering a career in GP before medical school (15.4%, IC 95% 7.8%-26.9%) was less than students who had completed the final year GP module (68.1%, IC 95% 55.9%-79.8%)  -There was a statistically significant difference in the number of students considering a career in GP before and after the FYGPM placement (p=0.008).  Influences on choice: Among students considering a career as GP, the perceptions that influenced the most were, in that order:  -GP lifestyle.  -Organization of GP.  -Approach to patient care.  -Other influences.  What influenced less was: community-based curriculum, positive media influences and dislike hospital careers.  Among students not considering a career as a GP, what influences most was:  -Other influences.  -Preference to hospital career.  -Dislike community-based curriculum, approach to patient care and GP organization.  What influences less was: the negative media influence and dislike GP lifestyle.  Influence of role models:  Outside the curriculum, both GPs and hospital doctors had negative and positive influences on individuals. Overall there was a neutral influence on students’ likelihood to prefer a career in general practice. |

# 7.Edgcumbe DP, Lillicrap MS, Benson JA. Scott. **A qualitative study of medical students’ attitudes to careers in general practice.** Education for primary care. 2008;19:65-73.

| **Author, year, country** | **Objective** | **Design** | **Participants** | **Quality** | **Commentaries** |
| --- | --- | --- | --- | --- | --- |
| Edgcumbe -2008.  UK | Examine the **views** about general practice (GP) as a potential career and the **influence** that have shaped them. | Qualitative methodology.  **Data collection:**  1) Individual semi-structured interviews.  Snowball sampling, iteratively until theoretical saturation.15 of 18 emailed students took part. Investigators generated a code list and it was sent to the participants who gave feedback.  **Analysis:** charting and mapping techniques in the framework system*.  2) Nominal group to triangulate data obtained from individual interviews. They generated headings and selected their top ten ideas for each category. Interviewed students were excluded. | Final year medical students at University of Cambridge.  N= 27  Interviews: 15 medical students (7male, 8 female).  Focus groups: 12 students (7 male, 5 female). | Moderate | **Strengths:**  -Two qualitative methods were used to triangulate the data and found little difference in the range of themes that each method identified, confirming theoretical saturation.  -Data were collected by a peer of the study participants.  **Limitations:**  -The fact that a peer collected the data may have influenced responses, but the language used by participants suggests that participants were speaking their minds directly.  **Conclusions:**  -Student’s experience is an important but not overriding factor in arriving at career decisions.  -Undergraduate experience in GP is important in allowing students to take a career choice from an informed position.  -Similar considerations had different effects on students. In general, training, income and work-life balance were attractive aspects.  **Comments**  -The heterogeneity of opinions held by students may be because the sampling method aimed to maximise diversity of opinion. |
| **RESULTS:** | | | | | |
| **Emerged themes in the interviews**  1-Experience of GP and of different medical careers during the clinical training influence students’ career intentions heavily: most had positive experiences, some had negative preconceptions and the experience diminish them but some had experiences that discouraged them.  2-GP as a work:  -Students whish to be free to choose a career.  -They draw a distinction between generalist and specialist work.  -Perception of GP to be a go-between, operating in a low-technology environment: prefer the acute rather than chronic medical conditions, prefer to deal with problems themselves without referral.  -Some students liked the variety of conditions seen by general physicians.  -Worried about perceived monotony.  -Business aspects of running a practice unappealing and some felt that the 2003 GP contract impinges on the professional autonomy  -Allow working anywhere but the prospect of remaining in one place put off some students.  -Concern about professional isolation.  -Anxiety for seeing the same patients regularly, or wanting quick answers in diagnosis.  -Concerns about managing risk.  -Holistic care: it was attractive for some students and not interesting for others.  3-GP training  -Short, well structured and flexible compared to hospital-based medicine.  -Competition in hospital training is unattractive.  -Research is not integrated in the training and this is perceived as positive for some and negative for others.  4-Work-life balance: easier to balance a career in GP with a family.  5-Income: some students consider GP well paid or overpaid particularly at earlier stages in their careers.  6-Status: the hospital-based specialists have a higher status than community-based generalist.  -they heard hospital doctors making derogatory comments about GP, and also GPs criticizing their hospital-based colleagues but in general they felt that this had not influenced them.  7-A second line option: GP is seen as an attractive option after an earlier hospital career. | | | | | |
| **Ranked views of participants of nominal groups**  Factors influencing career choice: 1-family/non-professional life, 2-intetest in area of work, 3-satisfaction with work “making a difference”, 4-individual aptitude for area of work, 5-flexible working (part-time, working hour choice), 6-on-call commitments, 7-working environment, 8-income, 9-pressure of job,10-length of training.  Positive aspects of GP as a potential career choice: 1-relationship with patients, 2-lifestyle, 3-flexible working arrangements, 4-range of cases, 5-working environment, 6-income, 7-special interests, 8-feeling part of the community, 9-public health, 10- independence.  Negative aspects of GP as a potential career choice: 1-mundane/repetitive work, 2-need to refer on, 3-administration, 4-isolation, 5-low status, 6-lack of time to spend with patients, 7-business management, 8-less team working, 9-new contract and government targets, 10-not very practical. | | | | | |

8.Chirk-Jenn NG, Kwok-Chi L, Cheong-Lieng T. **What do medical students think about primary care in Malaysia? A qualitative study**. Edu Prim Care.2005;16: 575-80.

| **Author, year, country** | **Objective** | **Design** | **Participants** | **Quality** | **Commentaries** |
| --- | --- | --- | --- | --- | --- |
| Chirk-Jenn, 2005.  Malaysia | To explore the **perceptions** of medical students towards primary care.  To understand the factors that influence their perceptions. | Qualitative methodology.  Exploratory study  **Data collection:**  Sampling method: snowballing.  -6 focus group discussions (3 in each university)  -Interactions among the participants. Field notes.  Focus group discussions were conducted until all researchers agreed that saturation of themes was achieved.  **Analysis:** Thematic analysis | 33 final year medical students from 2 universities in Malaysia (14 from the public university UM and 19 from the private university IMU^[[1]](#footnote-1)^).  These universities differ in fee structure, students’ socio-demographics, entrance examination results and ethnic distribution.  -Sex: 12 female, 21 males.  Mean age: 23,9 (range 22-27).  Nationalities: 15 Chinese, 10 Malays, 4 Indians and 4 other ethnic groups. | High | **Limitations:** possibility of researchers’ bias: researchers were involved in undergraduate teaching of this discipline. The facilitator of the focal groups was from a different university.  -Recruitment of participants at different stages of their clinical posting: those interviewed later would have more clinical experience and be more likely to integrate their knowledge and skills.  **Conclusions:** There are mixed perceptions of PC among medical students in Malaysia. Their attitudes seemed to be influenced by multiple factors: role modelling of tutors, lack of understanding of the discipline, the disparity between training and actual practice.  **Comments**-No differences in the opinions between participants from the two institutions, by gender, ethnicity or social background. |
| **RESULTS:**  1-Understanding of discipline  Holistic, comprehensive and patient-centred approach unique to primary care (+); It covered the breadth rather than depth of medicine;-Some lacked understanding equating GP to part of internal medicine or a combination of all other disciplines. A place to triage patients (differentiating the sick from the well); Some missed the action in the hospital; Bored by the repetition of common illnesses seen in GP;-Primary care posting is perceived as relaxed;-Opinions from colleagues and seniors influenced their perceptions; -It could be perceived as a discipline that teaches skills (communication skills, evidence-based medicine and counselling skills) rather than knowledge; Some found the principles of PC difficult to grasp like “time as a tool” and “doctor-patient rapport to modify behaviour”  2-Role modelling  Participants’ experience in their attachments to the GP in the community caused the strongest reaction: opposing views of how PC was practised in the “real word”. One student was putt of by the apparent lack of evidence-based practice and another was interested in family medicine because of his GP; Lecturers did not seem to influence on their perceptions;-Clinical knowledge and skills were important qualities; Doctor-patient relationships and communication skills were not highlighted.  3-The disparity between training and practice  What was taught in their classes was not practised in the community setting: time pressure, lack of support in solo private practice, difficulty in making decisions in a short consultation; Primary Care doctors in the government health centres were perceived to be less patient centred compared to the private GPs. | | | | | |

9.Firth A, Wass V. **Medical students’ perceptions of primary care: the influence of tutors, peers and the curriculum**. Edu Prim Care.2007;18:364-72.

| **Author, year, country** | **Objective** | **Design** | **Participants** | **Quality** | **Commentaries** |
| --- | --- | --- | --- | --- | --- |
| Firth, 2007.  UK | Explore views of undergraduate students on their **experiences of learning in primary care** in a curriculum with a strong community base. | Qualitative: phenomenology. Purposive sampling.  **Data collection:**  Semi-structured interviews: headings were identified from published literature and a brainstorming session with a final year group. A pilot interview was held. Interviews were taped and fully transcribed.  **Analysis:** iterative reading to identify and validate emergent themes using grounded theory. 2 independent observers cross checked 9 interviews to confirm the correction of transcription and that questioning was unbiased. 4 interviewees confirmed that the conclusions were valid. After 11 interviews, no new themes emerged. | 11 medical students (4 from 3rd year, 4 from 4th year, 3 from 5th year) of a medical school. Students were based in one of three teaching hospitals following the same problem-based curriculum.  Sex: 6 male, 5 female.  Mean age: 24,6 (range 20-37). | High | **Limitations:** findings may not generalise to other schools.  -Interviewer knew some of the interviewees although the study was carefully designed to ensure the sampling was truly purposive, the interview objectively delivered and the data cross validated to avoid subconscious bias.  -Differences in attitudes related to gender or ethnicity were not examined in depth.  -triangulation with other qualitative data, would have strengthened the findings.  **Strengths**: Saturation was reached after 11 interviews. Validation of the data confirmed it.  **Conclusions:** Students had strength negative preconceptions of GP on entry.  These preconceptions were reinforced within the medical school, not only by peers and tutors but also by the curriculum itself. |
| **RESULTS: EMERGED THEMES** | | | | | |
| **1-Student’s preconceived ideas of general practice (GP):** negative preconceptions based on:  -Personal experience: in general, negative ideas. The words “mundane” and “boring” were used when describing their views; The experience as a patient was one of the main reference points available; Some positive statements on the quality of care and relationships developed.  -The media portrayal of the profession as a major influence. Some programmes fostered the perception that GPs spent time “just chatting” and “not actually doing anything”. A minority o students viewed medical dramas as portraying general practice in an attractive light  -Early years in medical school: Students’ views were reinforced by tutors and peers. Many of their peers saw primary care in a negative light.  **2-Hierachical perceptions of GP both within the curriculum and from tutors:**  -Hospital tutors’ views influenced students’ perceptions of GP: they heard hospital doctors speaking about GPs in a detrimental way.  -The curriculum content appeared to influence students’ views: the majority of scenarios studied based within the hospital setting. Some students felt this added the notion that GP was less interesting than hospital medicine.  -Some students say all their peers found GP boring and an opportunity to take time off.  **3- Mixed experiences of placements**  -The majority of students held negative views of GP as a career, but developed positive views through their placements  -Recognition that an increasing amount of medical care was being carried out within primary care  -Benefit of being taught in primary care: opportunity to see cases not available in hospital and the “social side” of disease  -Range of case mix as a positive aspect and opportunity to work within the multidisciplinary team.  -The quality of the placement was the most influential factor, being the organisation and enthusiasm of the practice, and the GPs, the most beneficial determinants of a good placement. But there were several placements that involved just “sitting in the corner watching”. This damaged students’ perceptions of GP and tended to reinforce their preconceptions. Negative experiences were difficult to reverse.  **4-Reflections on future career choice and the Foundation programme^[[2]](#footnote-2)^.**  -Their attachments improved students’ views.  -GP offered a better lifestyle compared with hospital medicine (avoid out-of-hours duties and work part time) but at the undergraduate stage, this was not an important consideration.  -The business-driven nature of GP was seen as negative and stressful for 2 students and attractive to another.  -Several students recognised the benefits of an extended period in GP during the Foundation programme, but there was a concern that this would be very similar to the undergraduate community day.  -The quality and enthusiasm of the teachers were seen as vital in making Foundation training a success. | | | | | |

10.Mutha S, Takayama JI,O’Neil EH. Insights into medical students’ career choices based on third-and fourth-year students’ focus-group discussions

| **Author, year, country** | **Objective** | **Design** | **Participants** | **Quality** | **Commentaries** |
| --- | --- | --- | --- | --- | --- |
| Mutha, 1997, USA. | To identify beliefs and values that influence career decisions of medical students.  To characterize role models, assess educational experiences and financial considerations that influence career decisions. | Qualitative: phenomenology.  Random selection from class lists at each school (participation 70% of contacted).  **Data** **collection:** 12 focus groups (4 students each). Facilitators were medical students who had superior communication abilities and they were trained and assigned to focus groups on campuses other than their own and paid.  *When only one student kept the appointment, an individual interview was conducted (2 interviews were included)  *Two surveys to all participants: one before the discussion (19 items) for demographic information, and other post-discussion (4 items) to assess the effect of the group process on individual participation.  **Analysis:** audiotaped discussions were transcribed verbatim. Content and thematic analysis to determine patterns in responses and to identify and code frequently expressed ideas.  Triangulation comparing the results with other studies and reviewed with students, medical educators and medical school administrators. | 52 students from 3 medical schools in California.  3^rd^ year (24)  4^th^ year (28)  Mean age: 29 years (range: 23-41)  Sex: 27 women (52%)  Ethnicity: white 56%, Asian/pacific islander 23%, Latino 13%, African American 4%, other 4%.  Career preference of family medicine: 9 | High | **Limitations:** focus groups: students may be reluctant to discuss or identify influences that are incongruent with group expectations.  -Gender-specific issues were raised only when 50% or more of the participants were women.  **Strengths**: a confidential survey after the focus group: no student reported feeling inhibited but 4% did not discuss the career choices they had recorded in the pre-discussion surveys.  -Recall bias about decision making was addressed by scheduling discussions close in time to actual decisions and organizing discussions in temporal order: from entry into medical school, to clerkship experiences and to career decisions.  -Facilitators trained to use structured questions to minimize the likelihood that responses would be biased by how questions were asked.  -Triangulation process discovered the degree to which others experienced similar phenomena.  -Students from 3 schools with varying degrees of commitment to graduating generalist so the findings could have wider applicability  **Conclusions:** Medical effectiveness and knowledge mastery play important roles. Biases in the curricula and training settings may steer students away from generalist careers. Negative or absent role models revealed potential deficiencies in the recruitment and training of able faculty.  **Comments:** Initial analysis showed no thematic or content differences between focus groups and individual interviews; therefore, two interviews were included in the final analysis. |
| **RESULTS: EMERGED THEMES** | | | | | |
| 1-Effect through interventions or interactions  -Methods for achieving and measuring effect on patients differentiated the students interested in procedure-oriented fields from those interested in primary care. Students pursuing surgical specialties emphasized the achievement of immediate and tangible results, and students selecting primary care specialties desired continuous and long-term relationships with patients.  -Most of the students developed their perceptions about the effects of given fields during clinical rotations: inpatient services tended to discount the effects of cognitive specialties.  2-Scope of required knowledge: mastery of breadth  -They worried that the breadth of information required in generalist fields interfered with the ability to achieve competency and mastery and chose to narrow their focus by choosing subspecialty fields.  -For some students, primary care fields presented an intellectual challenge: an opportunity to address both a variety and complexity of medical problems.  -They discerned a gradient of difficulty in managing the knowledge base among generalist fields, with family medicine evoking the greatest concern.  3- Role models: positive, negative, or absent  -Clinicians (residents and attending physicians) influenced students’ career decisions.  -For some students, relationships with positive role models had influenced their specialty choices. Attributes of positive role models were: be a favourable persona, reputation in diagnostics skills, research, or teaching, and overt satisfaction with their careers.  -Exposure to positive role models was neither necessary nor sufficient for most of the students’ career decisions.  -Negative role models had strong dissuasive effects on specialty selections. This attributes were difficult personalities, perceived lack of camaraderie, professional dissatisfaction, and disheartening physician-patient interactions.  -The women sought, but could not identify role models. They attributed this to the lack of women in the field or their inability to identify with available individuals. The lack of role models deterred some women from considering particular fields and created anxieties and uncertainties.  4-The meaning of money  -Neither debt nor future income influenced students’ career decision.  -Many had difficulty conceptualizing potential income, especially relative to their current lifestyles.  -There were gender differences: women remarked that the anticipation of being in a dual-income family allowed them to minimize debt or income as a factor in their decisions. | | | | | |

1. UM: University of Malaya (public university). IMU: International Medical University (private) [↑](#footnote-ref-1)
2. The **Foundation Programme** is a two-year structured programme of workplace-based learning for junior doctors that forms a bridge between medical school and specialty/GP training. The programme aims to provide a safe, well-supervised environment for doctors to put into practice what they learned in medical school. It provides them with the generalist medical knowledge and skills to meet the requirements of the [General Medical Council](http://en.wikipedia.org/wiki/General_Medical_Council) (GMC) The New Doctor (2007) and the Foundation Programme Curriculum (2007) and prepares them for entry into specialty training. All medical graduates must undertake, and complete the Foundation Programme in order to work as a doctor in the UK. [↑](#footnote-ref-2)
